# SLO-2 Is Cytoprotective and Contributes to Mitochondrial Potassium Transport

**DOI:** 10.1371/journal.pone.0028287

**Published:** 2011-12-01

**Authors:** Andrew P. Wojtovich, Teresa A. Sherman, Sergiy M. Nadtochiy, William R. Urciuoli, Paul S. Brookes, Keith Nehrke

**Affiliations:** 1 Department of Medicine, University of Rochester Medical Center, Rochester, New York, United States of America; 2 Department of Anesthesiology, University of Rochester Medical Center, Rochester, New York, United States of America; 3 Department of Pharmacology and Physiology, University of Rochester Medical Center, Rochester, New York, United States of America; University of Houston, United States of America

## Abstract

Mitochondrial potassium channels are important mediators of cell protection against stress. The mitochondrial large-conductance “big” K^+^ channel (mBK) mediates the evolutionarily-conserved process of anesthetic preconditioning (APC), wherein exposure to volatile anesthetics initiates protection against ischemic injury. Despite the role of the mBK in cardioprotection, the molecular identity of the channel remains unknown. We investigated the attributes of the mBK using *C. elegans* and mouse genetic models coupled with measurements of mitochondrial K^+^ transport and APC. The canonical Ca^2+^-activated BK (or “maxi-K”) channel SLO1 was dispensable for both mitochondrial K^+^ transport and APC in both organisms. Instead, we found that the related but physiologically-distinct K^+^ channel SLO2 was required, and that SLO2-dependent mitochondrial K^+^ transport was triggered directly by volatile anesthetics. In addition, a SLO2 channel activator mimicked the protective effects of volatile anesthetics. These findings suggest that SLO2 contributes to protection from hypoxic injury by increasing the permeability of the mitochondrial inner membrane to K^+^.

## Introduction

Biological systems contain endogenous mechanisms for protection against stress. In particular, protection against ischemia-reperfusion (IR) injury is thought to proceed via opening of mitochondrial K^+^ channels [1]. Several cardioprotective strategies require such channels, and channel opening alone is sufficient to induce protection [2], [3]. For example, the protection by ischemic preconditioning involves the mitochondrial ATP-sensitive K^+^ (mK_ATP_) channel and activation of the channel is cardioprotective [2], [3], [4]. Similarly, volatile anesthetics protect the heart against IR injury, in a phenomenon termed “anesthetic preconditioning” (APC) [5], [6]. APC is evolutionarily conserved from *C. elegans* to mammals [7], and is potentially of clinical importance [6]. The precise mechanisms of APC remain elusive, although mitochondrial Ca^2+^ activated K^+^ channels have been proposed as mediators [8]. The canonical cell surface large-conductance, “big” K^+^ (BK) channel is coded for by the *slo-1* gene in worms and by the *Kcnma1* (*Slo1*) gene in mice. Pharmacologic evidence currently favors SLO1 as the mitochondrial BK (mBK) channel necessary for cardioprotection [9], [10].

However, the large-conductance K^+^ channel gene family also includes *Slo2*, represented by a single *slo-2* gene in worms and by two genes *Kcnt1* (*Slo2.2/Slack*) and *Kcnt2* (*Slo2.1/Slick*) in mice. Slo2 genes are widely expressed [11], [12], [13]. While a third Slo family member, *Slo3*/*Kcnu1,* has also been identified, its expression is restricted to mammalian spermatozoa [14]. The aim of this study was to combine the power of *C. elegans* genetics with mouse heart physiology and isolated mitochondrial assays, to investigate the relative contribution of SLO1 and SLO2 to mBK underlying APC. Here, we present evidence supporting an evolutionarily conserved role for SLO2 in facilitating mitochondrial K^+^ transport leading to APC.

## Methods

Full experimental details are in Supporting Information S1.

### C. elegans

Strains used in this study include Bristol-N2 (wild-type, WT); NM1968 *slo-1(js379)*V; LY100 *slo-2(nf100)*X; VC1819 *slo-2(ok2214)*X; KWN193 *pha-1(e2123)*III, *him-5(e1490)*V rnyEx112 [pKT60 (partial *Slo-2*: mCherry) recombined in vivo with linear WRM061CF07 fosmid, pCL1 (*pha-1*+)]; KWN 314 *slo-2(nf100)*X, *pha-1 (e2123ts)*III, rnyEx112; KWN 352 *pha-1(e2123)*III *him-5(e1490)*V, rnyEx216 [pKT111 (P*slo-2*:SLO-2:GFP), pCL1 (pha-1+)]. All mutant alleles were obtained from the *C. elegans* Genetic Center and were either obtained backcrossed or were backcrossed onto an N2-Bristol background. Single worm PCR genotyping was used to follow the mutant alleles.

### Mice

Characterization of the mBK channel was performed using male wild-type (WT) C57BL6 mice age 6–8 weeks purchased from The Jackson Laboratory (Bar Harbor, ME). Experiments involving *Slo1* ^-/-^ (KCNMA1^-/-^) knockout mice were on an FVB background [15]. Mice were genotyped by tail biopsy PCR, as previously described [15]. Male WT and *Slo1* ^-/-^ littermates age 6–8 weeks were used in experiments.

### Ethics Statement

All mice were maintained in an AAALAC-accredited pathogen-free barrier facility with food and water available *ad libitum*. All procedures were in accordance with the NIH Guide for the Care and Use of Laboratory Animals and were approved by an Institutional Animal Care and Use Committee (University Committee on Animal Resources (UCAR) protocol 2010–030).

### Hypoxic killing and APC of *C. elegans*

Experiments were performed on chronologically-synchronized populations of young-adult animals. Briefly, adult hermaphrodites were allowed to lay embryos for a period of two hours, and their adult progeny were tested for the ability to be preconditioned by 2% isoflurane or 4 hours of anoxia essentially as described [7].

### Isolation of mitochondria and BTC-AM loading

Mitochondria were isolated from *C. elegans* as previously described [16]. Mitochondria were isolated from mouse hearts as previously described [17]. The mitochondria were incubated with 20 µM BTC-AM and 0.05% Pluronic F-127 for 10 min at room temperature. The final mitochondrial pellet was suspended in 225 µl of the respective isolation medium described in Supporting Information S1 and stored on ice until use, within 1.5 hrs. Protein was determined by the Folin-phenol method [18].

### mBK thallium flux assay (Tl^+^-flux)

Tl^+^ is a surrogate for K^+^ and the Tl^+^-flux assay is widely used in the cellular K^+^ channel field [19]. Tl^+^ uptake into mitochondria were measured using a Varian Cary Eclipse spectrofluorometer as previously described [20] by monitoring changes in BTC fluorescence (λ_ex_ 488 nm, λ_em_ 525 nm).

### Mouse Langendorff *ex-vivo* perfused heart

Following anesthesia with avertin, a rodent 3-lead EKG was briefly obtained. A thoracotomy was then performed, and the aorta cannulated *in-situ* and rapidly transferred to a perfusion apparatus, as previously described [17]. The heart was perfused with Krebs-Henseleit buffer using constant flow and exposed to global ischemia, as detailed in Supporting Information S1. Following experimental protocols, hearts were stained and imaged as previously described to quantitate infarct size [21].

### Reagents

All chemicals were of the highest grade available from Sigma (St. Louis, MO) unless otherwise specified. Iberiotoxin, Charybdotoxin, and Apamin were from EMD Chemicals Group (Darmstadt, Germany); Bepridil and Paxilline were from Enzo Life Sciences International, Inc. (Plymouth Meeting, PA); Bithionol was from TCI America (Portland, OR); BTC-AM, Benzothiazole coumarin acetyoxymethyl ester was from Invitrogen (Carlsbad, CA).

### Statistics

Data presented are mean ± SEM. Statistical significance (P<0.05) between multiple groups was determined using analysis of variance (ANOVA). In whole worm studies, significance (P<0.05) was determined using a paired Student's *t*-test.

## Results

### Mouse heart mBK activity is insensitive to loss of *Slo1*

To investigate the role of *Slo* isoforms in conferring K^+^ transport across the mitochondrial inner membrane, a recently developed fluorescent assay [20] was applied to isolated mouse heart mitochondria, in which thallium (Tl^+^) flux serves as a K^+^ surrogate. Tl^+^, unlike K^+^, is not normally present in the mitochondrial matrix and so does not require depletion prior to the assay. In addition, Tl^+^ is transported very effectively by K^+^ channels. In conjunction with transporter-specific pharmacologic signatures, this technique allows for assessment of pathways that contribute to mitochondrial K^+^ transport. For example, we demonstrated previously that mK_ATP_ channels contribute to Tl^+^ flux [20].

**Figure 1:**
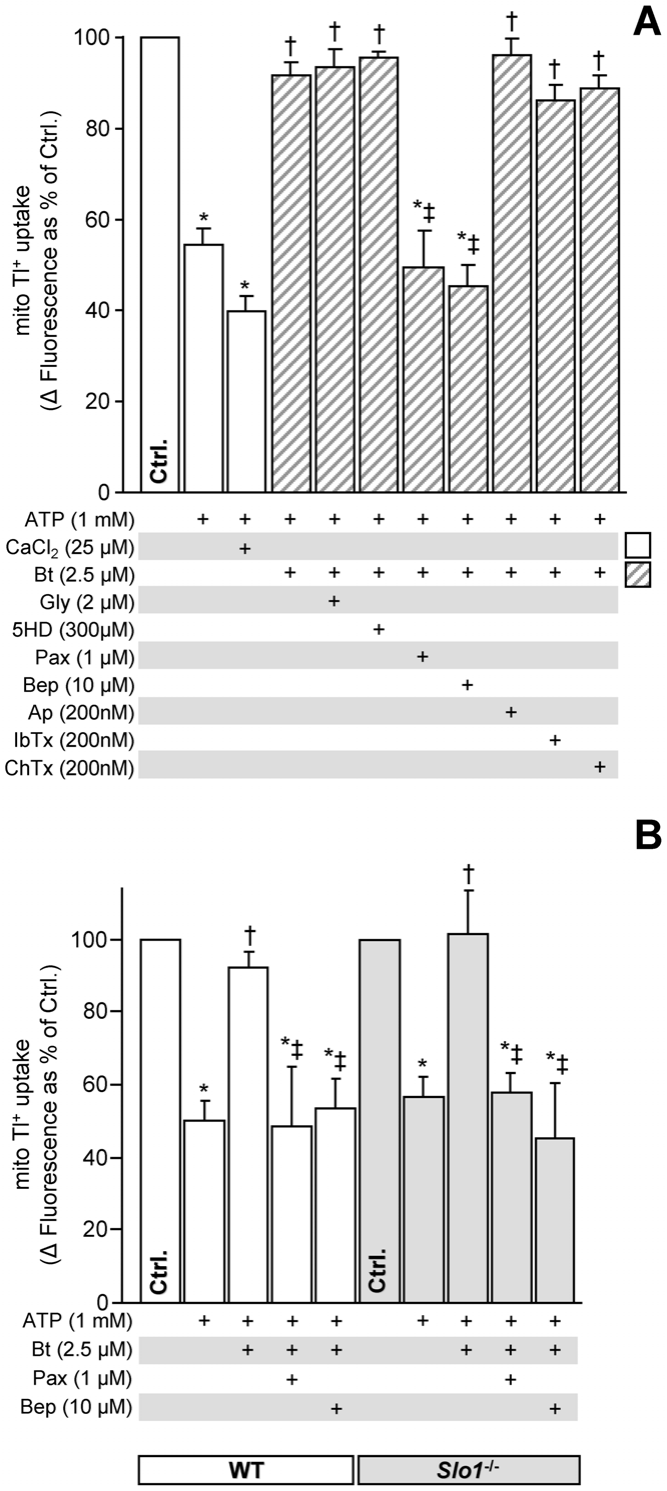
Mouse heart mBK channel activity is independent of *Slo1.* (A) Pharmacologic characterization of mBK activity in isolated WT (C57BL/6) mouse heart mitochondria, measured using the Tl^+^-flux assay. Mitochondrial thallium (Tl^+^) uptake, a surrogate for K^+^ flux, was measured using a mitochondrial matrix loaded Tl^+^-sensitive fluorophore, as detailed in the methods section of Supporting Information S1. ATP was added to block the normally open mitochondrial mK_ATP_ channel [38], [20], [16]. Graph shows Δ fluorescence upon Tl^+^ addition to media. Where indicated, the BK channel activators Ca^2+^ (open bars) or bithionol (Bt, hatched bars) were added at the concentrations indicated. Optimal doses are shown, since higher concentrations compromised mitochondrial function (data not shown). The mK_ATP_ inhbitors glyburide (Gly) and 5-hydroxydecanoate (5-HD), and the K^+^ channel inhibitors paxilline (Pax), bepridil (Bep), apamin (Ap), charybdotoxin (ChTx) and iberiotoxin (Ibtx) were present as listed below the x-axis. (B) mBK channel activity in heart mitochondria isolated from *Slo1^-/-^* and WT FVB littermate mice. The baseline Δ fluorescence (Ctrl, set to 100%) was 21±4 and 22±4 arbitrary units in WT (white bars) and *Slo1^-/-^* (gray bars) respectively. Pharmacologic agents were present as listed below the x-axis. Data are means ± SEM, N>3 (N = independent mitochondria preparations). *p<0.05 vs. ctrl, †p<0.05 vs. ATP, ‡p<0.05 vs. ATP+Bt.

The mBK is a critical component of APC-mediated cardioprotection [8], and to date the *Slo1* gene product has been thought to underlie mBK activity [9], [10]. Under conditions where ATP was present to block mK_ATP_ channels, we observed mitochondrial Tl^+^ flux that was activated by bithionol and inhibited by paxilline (Figure 1A), consistent with a BK channel activity [11], [22]. However, Tl^+^ flux was not affected by iberiotoxin, charybdotoxin or apamin (Figure 1A). Apamin is specific for small-conductance K^+^ channels, and so this result was not surprising. However, charybdotoxin and iberiotoxin are thought to target the canonical BK channel *Slo1*, which is voltage and calcium-activated. In light of this observation, we also tested the ability of Ca^2+^ to activate the Tl^+^ transport activity and found it to be ineffective (Figure 1A). Such Ca^2+^ insensitivity has been previously reported for cardiac mitochondrial K^+^ fluxes [23]. Finally, we found that bepridil, which targets voltage activated Ca^2+^ channels and K_Na_ channels [22], inhibited mBK (Figure 1A). Paxilline, Ca^2+^, and bepridil did not disrupt the membrane potential (Table S1).

Based on these observations, we obtained a mouse strain containing the *Kcnma1^tm1Rwa^* allele targeting *Slo1* (referred to hereafter as *Slo1^-/-^*) [15] and investigated mBK activity (biothionol-activated Tl^+^ transport with the above described pharmacologic sensitivities) in purified heart mitochondria. The results (Figure 1B) show an identical mBK activity and pharmacologic profile in both *Slo1* ^-/-^ and control wild-type (WT) littermates, suggesting mBK is not *Slo1*-derived.

### Anesthetic preconditioning of perfused mouse hearts is insensitive to loss of *Slo1*

**Figure 2:**
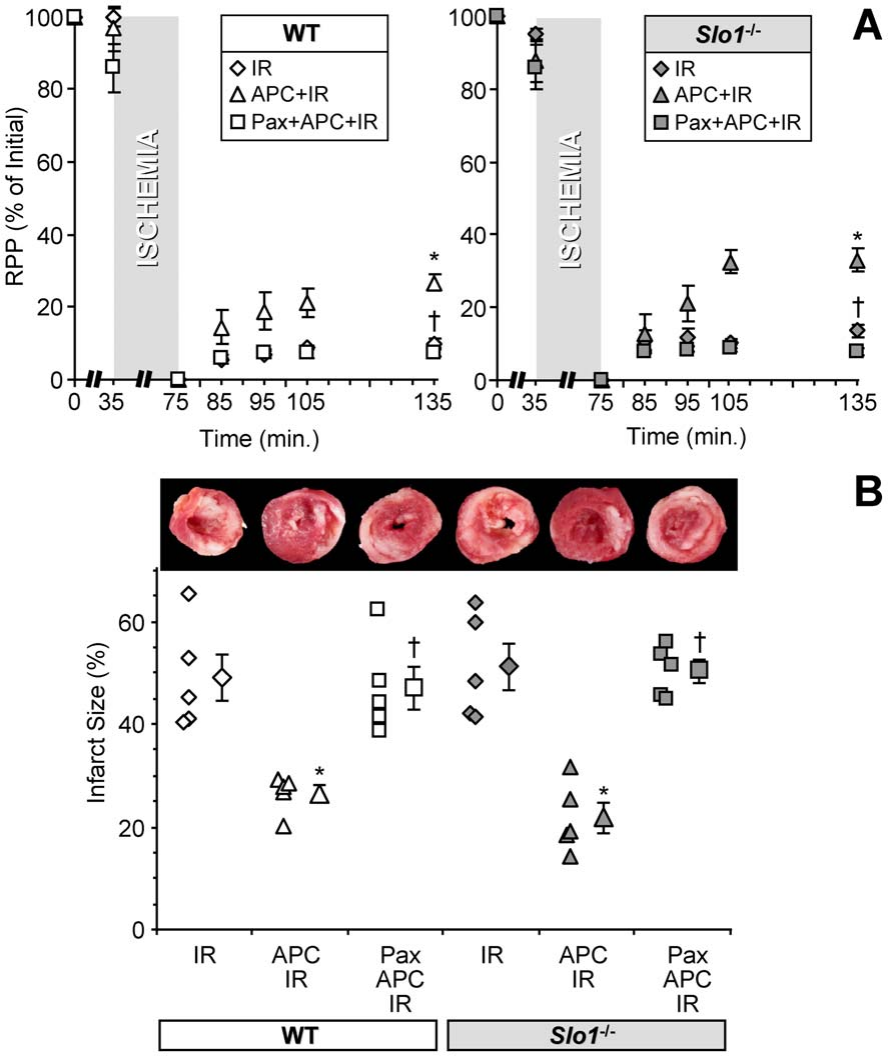
APC in mouse hearts is independent of *Slo1.* Perfused hearts were subjected to IR injury, APC+IR, or paxilline (Pax)+APC+IR, as outlined in the methods section of Supporting Information S1. (A) Left-ventricular function (heart rate x pressure product, RPP) was monitored throughout, and is expressed as % of initial value. Data for WT (white symbols) and *Slo1^-/-^* (gray symbols) FVB littermates are shown on separate axes for clarity. (B) Upon completion of IR protocols, hearts were sliced, fixed and stained with TTC, to delineate live (red) and infarcted (white) tissue. Upper panel shows typical slices used for quantitation of infarct area. Lower panel shows infarct expressed as a percent of the area at risk (100% in this global ischemia model). Data are means ± SEM, N = 5 (N = independent hearts) *p<0.05 vs. IR, †p<0.05 vs. APC+IR.

Since mBK is thought to contribute to APC, we next tested whether *Slo1* contributes to protection against IR injury by the volatile anesthetic isoflurane. APC protection was measured in *ex-vivo* perfused hearts from *Slo1* ^-/-^ mice and control WT littermates. As previously reported [24], *Slo1* ^-/-^ mice exhibited a slightly lower heart rate (Table S2). Nevertheless, both strains exhibited identical sensitivity to baseline IR injury (infarct size, and recovery of rate x pressure product) (Figure 2 and Table S3). Furthermore, no difference in APC-induced protection was observed between *Slo1* ^-/-^ and WT hearts (Figure 2 and Table S3), suggesting that protection by APC is SLO1-independent. Moreover, protection by ischemic preconditioning (IPC) was preserved in *Slo1* ^-/-^ mice (Figure S1 and Table S3). Although these results suggest that SLO1 channel activity is not necessary for APC or IPC, they do not negate the idea that SLO1 may be an important pharmacologic target for cardioprotection, or may be involved in other protective mechanisms [9].

We note that APC-induced cardioprotection was ablated by paxilline in both the *Slo1^-/-^* and WT hearts (Figure 2), though paxilline alone had no effect on baseline IR injury (Figure S2). This data suggests that an alternate paxilline-sensitive transporter might be involved in APC. The SLO3 channel is unlikely to account for the observed K^+^ transport activity as it is relatively insensitive to paxilline [25] and its expression is restricted to testes. On the other hand, both the *Slo2.1* (Slick) and *Slo2.2* (Slack) mammalian *Slo2* paralogues are widely expressed. Furthermore, they have been shown to be Ca^2+^ insensitive [26], [27], [28], which is in agreement with the mitochondrial Tl^+^ flux data (Figure 1).

SLO2 channels are instead activated by Na^+^, and while this would be an intriguing characteristic to test, using Na^+^ to stimulate Tl^+^ uptake is problematic as Na^+^ affects mitochondrial function. Initial experiements with cardiac sub-cellular protein fractions revealed an immuno-reactive band in the mitochondria using both anti-*Slo2.1* and anti-*Slo2.2* antibodies that was close to the predicted molecular weights of Slo2.1 and Slo2.2 proteins (Figure S3). The presence of additional bands in cardiac homogenates, however, precluded the use of these antibodies for immunohistochemical protein localization in fixed tissues. Likewise, using genetics to test the role of SLO2 in mice is complicated by the fact that, unlike *Slo1* (and *Slo3*), neither of the *Slo2* deletion strains have yet been reported. For this reason, we turned to the genetic model organism *C. elegans*.

### The *slo-2* gene product contributes to anesthetic preconditioning in *C. elegans*

**Figure 3:**
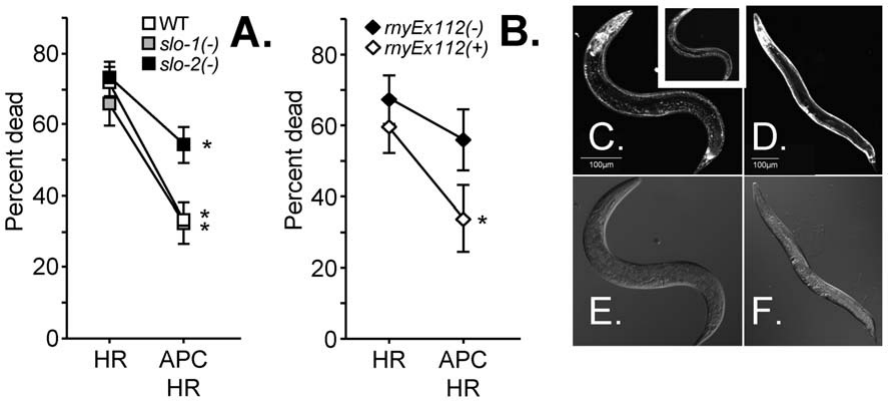
APC in *C. elegans* requires *slo-2.* (A) *C. elegans* WT control (N2-Bristol), *slo-1(js379)*V and *slo-2(nf100)*X mutants were subjected to hypoxia-reoxygenation (HR) and isoflurane APC+HR, as detailed in the methods section of Supporting Information S1. Viability is expressed as percent of dead worms. Means ± SEM, N>11, (N = independent trials of >100 worms per trial), *p<0.05 vs. HR. (B) HR and APC+HR of fluorescent *slo-2(nf100)*X mutant *C. elegans* containing an extrachromasomal array derived from a mixture of a SLO-2:mCherry gene fusion and the WRM061CF07 fosmid (rnyEx112(+)) compared to their non-fluorescent siblings that had lost the array (rnyEx112(-)). Means ± SEM, N = 4 (N = independent trials of >100 worms per trial), *P<0.05 vs. HR. (C–F) Fluorescent confocal maximum projection (C, D) and corresponding DIC (E, F) images of *C. elegans* expressing SLO-2:mCherry (C, E) or SLO-2:GFP protein fusions (D, F) in neurons and muscle via the native *slo-2* promoter. The white inset in panel C shows a single confocal slice obtained close to the surface of the worm, showing clear body wall muscle plasma membrane localization of the mCherry fusion protein.

APC is known to protect the nematode *C. elegans* from subsequent hypoxic death [7], and worm orthologs of *Slo1* and *Slo2* exist. Further simplifying the use of this model is the fact that the worm *slo-2* gene has not diverged into *Slo2.1* and *Slo2.2* paralogs. Since loss-of-function mutants of both *slo-1* and *slo-2* are viable in worms, to determine which gene products are required for APC, these mutants were tested for their ability to be preconditioned by isoflurane. As shown in Figure 3A, APC significantly improved the resistance of both wild-type (WT) and *slo-1* ablated worms to hypoxic stress. This result matched our findings with the mouse *Slo1* knockout model (Figure 2). However, the loss of *slo-2* largely blocked protection by APC (Figure 3A). These results were confirmed by using a second distinct *slo-2* mutant allele (Table S4). We further demonstrated that reintroducing the native *slo-2* gene (contained in the WRM061CF07 fosmid) into the *slo-2* mutant could restore the beneficial effects of APC (Figure 3B). A transgenic fusion of SLO-2 to either mCherry or GFP (Figure 3C–F) was unable to rescue the mutant (data not shown), suggesting that the “tagged” protein is either non-functional or does not localize correctly. Remarkably, all strains were protected by IPC (Figure S4) emphasizing the specificity of *slo-2* for APC-mediated protection.

Unlike several recently identified transporters whose activity is restricted to the mitochondrial inner membrane [29], [30], [31] SLO-2 was not expected to localize to the mitochondria exclusively. As has been pointed out for the mK_ATP_ channel [32], there may only be a few molecules per mitochondrion, particularly given SLO2's large unitary conductance, and confocal analysis of the SLO-2 fluorescent protein fusions indicated mainly plasma membrane localization (data not shown). In short, while these results confirmed that *SLO2* contributes to effective APC in the worm model, they did not rule out a role for a plasma membrane, rather than mitochondrial, conductance in this process. This prompted us to investigate the role of *Slo* isoforms in conferring K^+^ transport across the mitochondrial inner membrane in worms more directly.

### mBK channel activity *C. elegans* requires *slo-2*

**Figure 4:**
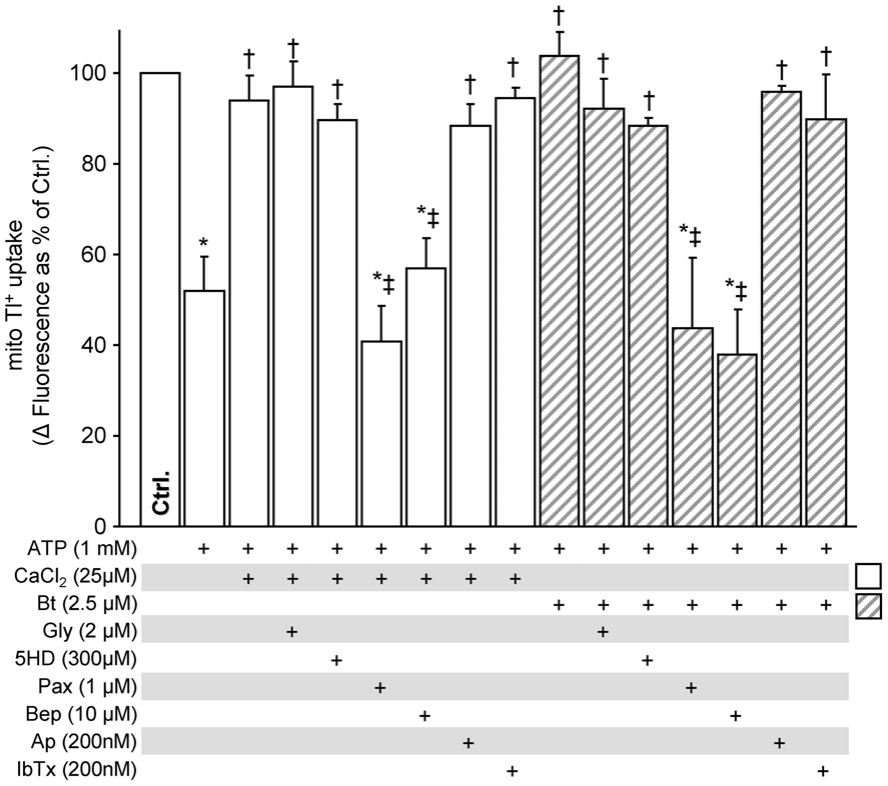
Pharmacologic characteristics of *C. elegans* mBK channel activity in the wildtype N2-Bristol strain. Characterization of mBK activity in isolated WT (N2-Bristol) worm mitochondria was measured using the Tl^+^-flux assay as in Figure 1. Mitochondrial Tl^+^ uptake, a surrogate for K^+^ flux, was measured using a mitochondrial matrix loaded Tl^+^-sensitive fluorophore, as detailed in the methods section of Supporting Information S1. ATP was added to block the normally open *C. elegans* mitochondrial mK_ATP_ channel [16]. Graph shows Δ fluorescence upon Tl^+^ addition to media. Channel activators and inhibitors were added as defined in Figure 1, and listed below the x-axis. Data are means ± SEM, N>4 (N = independent mitochondria preparations). *p<0.05 vs. ctrl, †p<0.05 vs. ATP, ‡p<0.05 vs. ATP+Bt.

mBK activity was measured in isolated *C. elegans* mitochondria using the same Tl^+^ flux assay described above for mouse mitochondria [16]. *C. elegans* mitochondria exhibited an ATP-insensitive Tl^+^ flux that was activated by the BK agonist bithionol, insensitive to the mK_ATP_ blockers 5-hydroxydecanoate and glyburide, and blocked by the BK channel inhibitor paxilline and by bepridil (Figure 4). Both paxilline and bepridil did not decrease the Tl^+^ flux signal below the signal of ATP alone (ATP+Pax, 57.8±13.1; ATP+Bep, 51.7±11.4). These pharmacologic properties fit the profile of a classical BK channel [33], [22], [34]. Interestingly, unlike in mice, Ca^2+^ was capable of activating the mBK in worms (Figure 4); the *slo-2* gene product in worms is sensitive to Ca^2+^ [35]. Notably, the *C. elegans* mBK was insensitive to the SLO-1 inhibitors iberiotoxin and charybdotoxin as well [11], [12].

**Figure 5:**
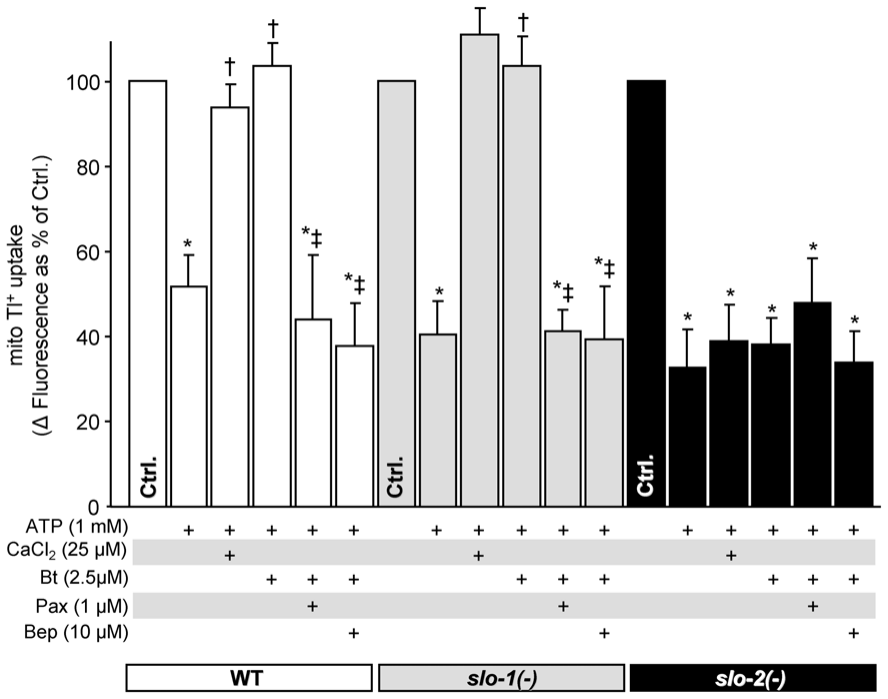
*C. elegans* mBK channel activity requires *slo-2.* mBK channel activity in WT (N2-Bristol; white bars), *slo-1* (js379; gray bars) and *slo-2* (nf100; black bars) *C. elegans*. The baseline Δ fluorescence (Ctrl, set to 100%) was 29±6, 31±4 and 26±4 arbitrary units in WT, *slo-1* and *slo-2*, respectively. Pharmacologic agents were present as listed below the x-axis. Data are means ± SEM, N>4 (N = independent mitochondria preparations). *p<0.05 vs. ctrl, †p<0.05 vs. ATP, ‡p<0.05 vs. ATP+Ca^2+^ or ATP+Bt.

Measuring mBK activity in mitochondria from WT, *slo-1* and *slo-2* mutants (Figure 5) revealed that only loss of *slo-2* abolished bithionol-stimulated Tl^+^ flux. This effect could not be attributed to a general effect on mitochondrial function, since mK_ATP_ channel activity was not different between strains. Overall, these data suggest that *slo-2* expression contributes to both mBK conductance and APC in *C. elegans*, while *slo-1* is dispensable for both.

### The mBK channel is activated by isoflurane, and pharmacologic channel activation is sufficient to elicit hypoxic protection

**Figure 6:**
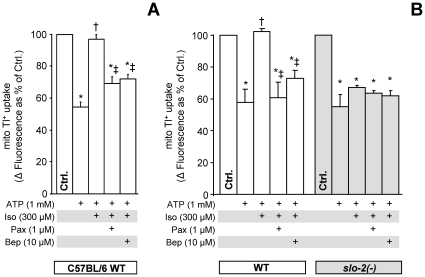
Isoflurane activation of mBK channel activity in mouse and *C. elegans.* Mitochondrial Tl^+^ uptake was measured using a mitochondrial matrix loaded Tl^+^-sensitive fluorophore, as detailed in the methods section of Supporting Information S1. ATP was added to block the normally open mK_ATP_ channel. Characterization of mBK channel activity in isolated mitochondria from (A) WT (C57BL/6) mouse hearts and (B) WT (N2 Bristol) and *slo-2*(*nf100*) mutant *C. elegans*. Graph shows Δ fluorescence upon Tl^+^ addition to media. Where indicated, isoflurane (Iso) and the K^+^ channel inhibitors paxilline (Pax) and bepridil (Bep) were present as listed below the x-axis. Data are means ± SEM, N≥3 (N = independent mitochondria preparations). *p<0.05 vs. ctrl, †p<0.05 vs. ATP, ‡p<0.05 vs. ATP+Iso.

Currently there is no evidence for mBK activation by volatile anesthetics. In Figure 6 we demonstrate that isoflurane caused an increase in Tl^+^ flux through the mitochondrial inner membrane in both mice and worms. The pharmacologic characteristics of the Tl^+^ transport activity are identical to those observed for the bithionol-activated channel in both species (i.e., blocked by paxilline and bepredil). Furthermore, isoflurane-stimulated channel activity was ablated in mitochondria from *slo-2* mutant worms (Figure 6B).

**Figure 7:**
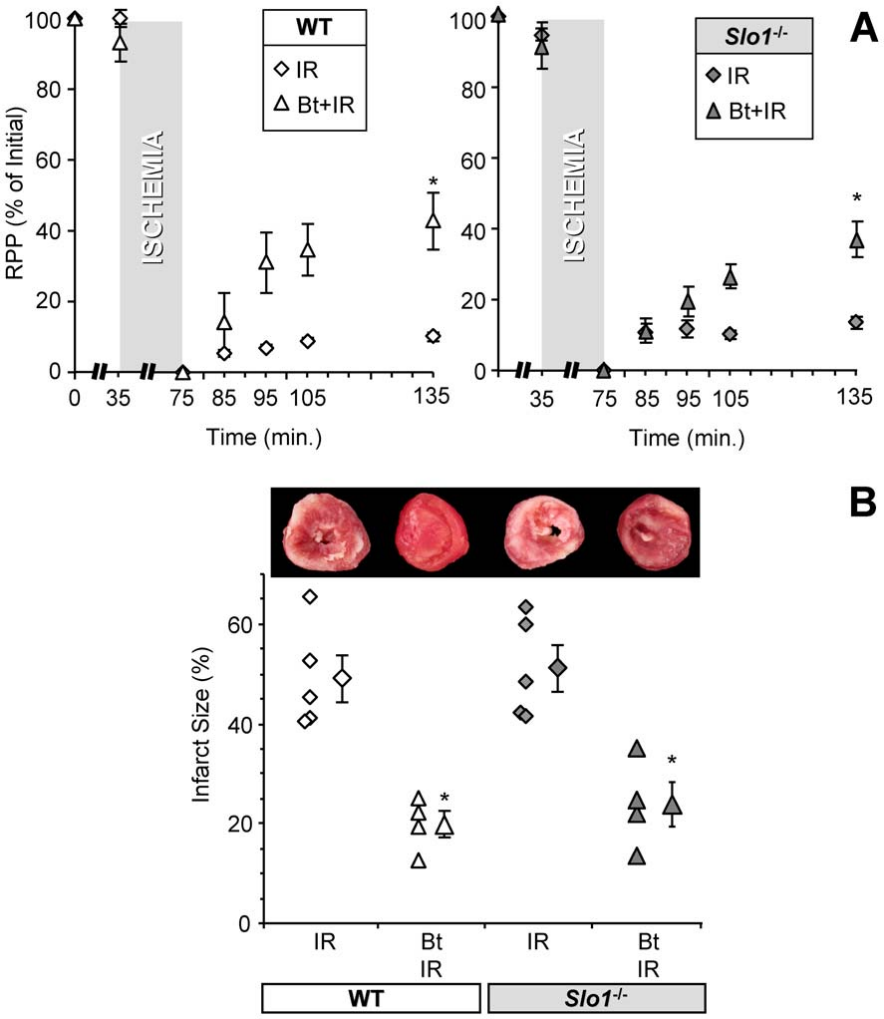
Cardioprotection by the BK activator bithionol. Perfused hearts were subjected to IR injury (from Figure 2) or Bithionol (Bt)+IR in WT (white symbols) and *Slo1^-/-^* (gray symbols) FVB littermate mice, as outlined in Supporting Information S1 methods. (A) Left-ventricular function (heart rate x pressure product, RPP) was monitored throughout, and is expressed as % of initial value. (B) Upon completion of IR protocols, hearts were sliced, fixed and stained with TTC, to delineate live (red) and infarcted (white) tissue. Upper panel shows typical slices used for quantitation of infarct area. Lower panel shows infarct expressed as a percent of the area at risk (100% in this global ischemia model). All data are means ± SEM, N≥4 (N = independent hearts). *p<0.05 vs. IR.

Finally, although our results suggest that *Slo2* channel opening by volatile anesthetics is necessary for full protection by APC, we wanted to determine whether channel opening is sufficient to recapitulate the protective effect of APC. Thus, the pharmacologic BK activator bithionol was tested for its ability to protect hearts from IR injury in the mouse heart perfusion model. The data indicate that bithionol pre-treatment elicits significant protection as reflected by reduced infarct size, and enhanced recovery of rate x pressure product (Figure 7). Moreover, the protection afforded by bithionol pretreatment was retained in *Slo1^-/-^* hearts (Figure 7), confirming that the SLO1 channel is not the physiologic target of bithionol that elicits protection. These data suggest that opening of a BK channel, most likely mitochondrial and derived from *Slo2*, is both necessary and sufficient for cardioprotection from ischemia.

## Discussion

Several mitochondrial K^+^ channel sub-types have been implicated in protection against ischemic injury [1], yet to date all such channels remain undefined at the molecular level. Overall, the unique combination of genetics and pharmacologic signatures studied herein are consistent with a conclusion that SLO2 underlies mBK activity and APC-induced protection in both *C. elegans* and perhaps in mouse hearts, as well.

While the mBK channel in both *C. elegans* and mouse mitochondria displayed similar pharmacological profiles, only the *C elegans* channel was activated by Ca^2+^. These results are consistent with the observation that *C elegans* SLO-2 channels are activated by Ca^2+^, while the mammalian Slo2 ortholog is instead activated by Na^+^ [28], [26], and support the hypothesis that the mBK necessary for mitochondrial K^+^ transport and APC is derived from SLO2 in both *C elegans* and mice. It is interesting that *C. elegans* lack a voltage-gated Na^+^ channel [36]. The differing ion-sensitivities between mammals and *C. elegans* Slo2 channels may reflect underlying differences in ischemic electrolyte handling [26], and in this respect, it is noteworthy that the Slo2 channel characteristics appear to reflect this divergence.

It is not yet clear which mammalian SLO2 paralogue may constitute the mBK channel. In fact, both SLO2.1 and SLO2.2 may play important roles, since the optimal sub-cellular localization of each paralogue appears to depend on formation of heteromers with the other [37], [12]. Unfortunately, there are currently no pharmacologic tools to distinguish between SLO2.1 and SLO2.2, and although SLO2.1 is blocked by ATP [11] this effect is overridden by pharmacologic channel activators (similar to mK_ATP_ [38], [20]), and it is not known how SLO2 heteromer formation impacts ATP sensitivity.

While in *C. elegans slo-2* was not necessary for protection by IPC, from the perspective of ischemia however, we find it interesting that SLO2.2 is activated by hypercapnia and acidosis [27] both which protect against IR injury [39], [40], [41]. Of particular interest is the role of acidosis in activating SLO2 since alkalinization of the matrix is a result of an open mitochondrial K^+^ channel [42] and represents a possible mechanism of feedback inhibition. Likewise, the observation that both SLO2.1 and SLO2.2 channels exhibit K_Na_ activity [11], [27] may have implications for mammalian mBK channel activity during ischemia, in which intracellular Na^+^ is elevated [43], [44]. These commonalities may warrant further investigation into the role of SLO2.1 and SLO2.2 in IPC in a mammalian context.

It is clear from our data that SLO1 is not required for either APC or IPC in worms or in mice. However, our results do not preclude the presence of SLO1 in the mitochondria or a role for SLO1 channels in other protective paradigms. In fact, the *Slo* family of K^+^ channels exhibits a wide diversity of auxiliary subunits and multimeric assemblies in different tissues [37], yielding diverse activities, ion/second-messenger sensitivities, and roles in cellular processes [26]. In particular, SLO1 and SLO2.2 can heteromultimerize yielding a channel with intermediate properties [12]. For example, SLO1 channel is sensitive to iberiotoxin and SLO2.2 is insensitive to Ca^2+^ but a SLO1/SLO2.2 heteromultimer channel is insensitive to iberiotoxin and sensitive to Ca^2+^ [12]. Thus, the tissue diversity in BK composition may explain previous pharmacology-based studies suggesting a *Slo1*-derived mBK in other tissues [10]. Similarly, *slo-1* in worms is known to regulate neurotransmitter release at the synaptic junction [45] where it acts upstream or parallel to syntaxin [46]. Mutants in either of these genes can alter the effect of anesthetics on behavior [46]. It is possible that *slo-1* acting through this pathway could indirectly affect mitochondrial function through humoral neurosecretory mechanisms. However, our unpublished results confirm observations that syntaxin mutants, like *slo-1* mutants, are nevertheless able to be effectively preconditioned by isofluorane [7].

While it is possible that mBK activation may occur directly through anesthetic stimulation, and we have demonstrated that mBK in isolated mitochondria are opened by isofluorane (Figure 6), it is also possible that activation occurs indirectly via other anesthetic targets such as protein kinases that may co-purify with mitochondria [8], [47], [48]. Such activation would then stimulate mitochondrial K^+^ uptake, with subsequent protection from ischemia occurring via downstream mechanisms that are not yet clear. The activation of mitochondrial K^+^ channels is proposed to mediate protection via the regulation of the mitochondrial matrix volume, decreasing mitochondrial Ca^2+^ uptake and modulating reactive oxygen species generation [4]. Through these events activation of the mBK might block the formation of the mitochondrial permeability transition pore and subsequent cell death [4].

In conclusion, the *slo-2* gene in worms contributes to a K^+^ influx pathway at the mitochondrial inner membrane with a nearly identical pharmacologic sensitivity to a repertoire of activators and inhibitors as recombinant SLO2 channels. Furthermore, *slo-2* is necessary for APC-induced protection in worms. Conversely, SLO1 does not contribute to the mitochondrial K^+^ influx pathway studied here, nor is it required for APC or IPC in either worms or mice. We hypothesize that given these results, *Slo2* is likely the APC-relevant channel in mice, and our results further suggest that pharmacologic opening of *Slo2* elicits protection. These results have the potential to refocus the design of anti-ischemic therapeutics.

## Supporting Information

Figure S1 **IPC in mouse hearts is independent of** ***Slo1.*** Perfused hearts were subjected to IR injury (from Figure 2) or ischemic preconditioning (IPC)+IR, as outlined in Supporting Information S1 methods. (A) Left-ventricular function (heart rate x pressure product, RPP) was monitored throughout, and is expressed as % of initial value. Data for WT (white symbols) and *Slo1^-/-^* (gray symbols) FVB littermates are shown on separate axes for clarity. (B) Upon completion of IR protocols, hearts were sliced, fixed and stained with tetrazolium chloride, to delineate live (red) and infarcted (white) tissue. Upper panel shows typical slices used for quantitation of infarct area. Lower panel shows infarct expressed as a percent of the area at risk (100% in this global ischemia model). All data are means ± SEM, N≥4 (N = independent hearts).*p<0.05 vs. IR.
(TIF)

Figure S2 **Paxilline in mouse hearts does not affect ischemic sensitivities.** Perfused hearts were subjected to IR injury (from Figure 2) or paxilline (Pax)+IR, as outlined in Supporting Information S1 methods. (A) Left-ventricular function (heart rate x pressure product, RPP) was monitored throughout, and is expressed as % of initial value. Data for WT (white symbols) and *Slo1^-/-^* (gray symbols) FVB littermates are shown on separate axes for clarity. (B) Upon completion of IR protocols, hearts were sliced, fixed and stained with tetrazolium chloride, to delineate live (red) and infarcted (white) tissue. Upper panel shows typical slices used for quantitation of infarct area. Lower panel shows infarct expressed as a percent of the area at risk (100% in this global ischemia model). All data are means ± SEM, N = 5 (N =  independent hearts).
(TIF)

Figure S3 **Immunoblot analysis of SLO2 in fractionated cardiac tissue.** Homogenate from WT (C57BL/6) mouse hearts was fractionated and the proteins were separated by SDS-PAGE. Slo2.1 and Slo2.2 were detected by immunoblot analysis (NeuroMab antibodies), as detailed in Supporting Information S1 methods. Western blots for GAPDH, adenine nucleotide translocator 1 (ANT1) and histones validated separation of the homogenate into cytosolic, mitochondrial and nuclear fractions, respectively.
(TIF)

Figure S4 **IPC in** ***C. elegans*** **is independent of** ***slo-1*** **and** ***slo-2.*** WT, *slo-1(js379)* and *slo-2(nf100)* mutants were subjected to hypoxia-reoxygenation (HR) and ischemic preconditioning IPC+HR, as detailed in the methods section of Supporting Information S1. Viability is expressed as percent of dead worms. Means ± SEM, N = 4 (N = independent trials of >100 worms per trial), *p<0.05 vs. HR.
(TIF)

Table S1 **Mitochondrial membrane potential is not affected by channel modulators.** Mitochondria were isolated from WT (C57BL/6) mice and loaded with a fluorescent indicator (TMRE 20 nM or JC-1 0.2 µg/mL) in the presence of either Bithionol (2.5 µM), CaCl_2_ (25 µM), Paxilline (1 µM) or Bepridil (10 µM). Fluorescent indicators accumulate in mitochondria in relation to membrane potential (Δψ_m_). Following stabilization, Δψ_m_ was collapsed via addition of Δψ_m_ FCCP (10 µM) resulting in a re-distribution of the fluorescent indicator, resulting in a decrease in fluorescence. All data are means ± SEM, N≥3 and are not significantly different (N = independent mitochondria isolation of ≥3 mouse hearts).
(PDF)

Table S2 **EKG parameters of Avertin anesthetized wild-type (WT) and** ***Slo1*** **^-/-^ littermate FVB mice**. EKG was collected as outlined in the methods. All data are means ± SEM, N≥13 *p<0.05 vs. WT.
(PDF)

Table S3 **Mouse langendorff functional parameters.** Groups are indicated in the left column in both wild-type (WT, top table) and *Slo1* ^-/-^ (bottom table) FVB littermates. Parameters (indicated in the top row) were measured at the time points indicated. LVDP, left ventricular developed pressure (% of control). Data are means ± SEM, N≥4 *p<0.05 vs. IR (60 min Reperfusion). †p<0.05 vs. APC+IR (60 min Reperfusion).
(PDF)

Table S4 **APC-dependent protection in** ***C. elegans*** **requires** ***slo-2.*** *C. elegans* WT control (N2-Bristol), *slo-1(js379)*V, *slo-2(nf100)*X, and *slo-2(ok2214)*X mutants were subjected to hypoxia-reoxygenation (HR) and isoflurane APC+HR, as detailed in the methods section of Supporting Information S1, and the average reduction in % death was determined. Means ± SEM. N2, N = 28; *slo-1(js379)*, N = 12; *slo-2(nf100)*, N = 27; *slo-2(ok2214)*, N = 4. (N =  independent trials of >100 worms per trial).
(PDF)

Supporting Information S1 **Supplementary methods.**
(DOC)
