# Supplementary figures and images for: SLO-2 Is Cytoprotective and Contributes to Mitochondrial Potassium Transport
Source: PLoS One. 2011 Dec 1;6(12):e28287. doi: 10.1371/journal.pone.0028287 (PMC3228735; doi:10.1371/journal.pone.0028287)

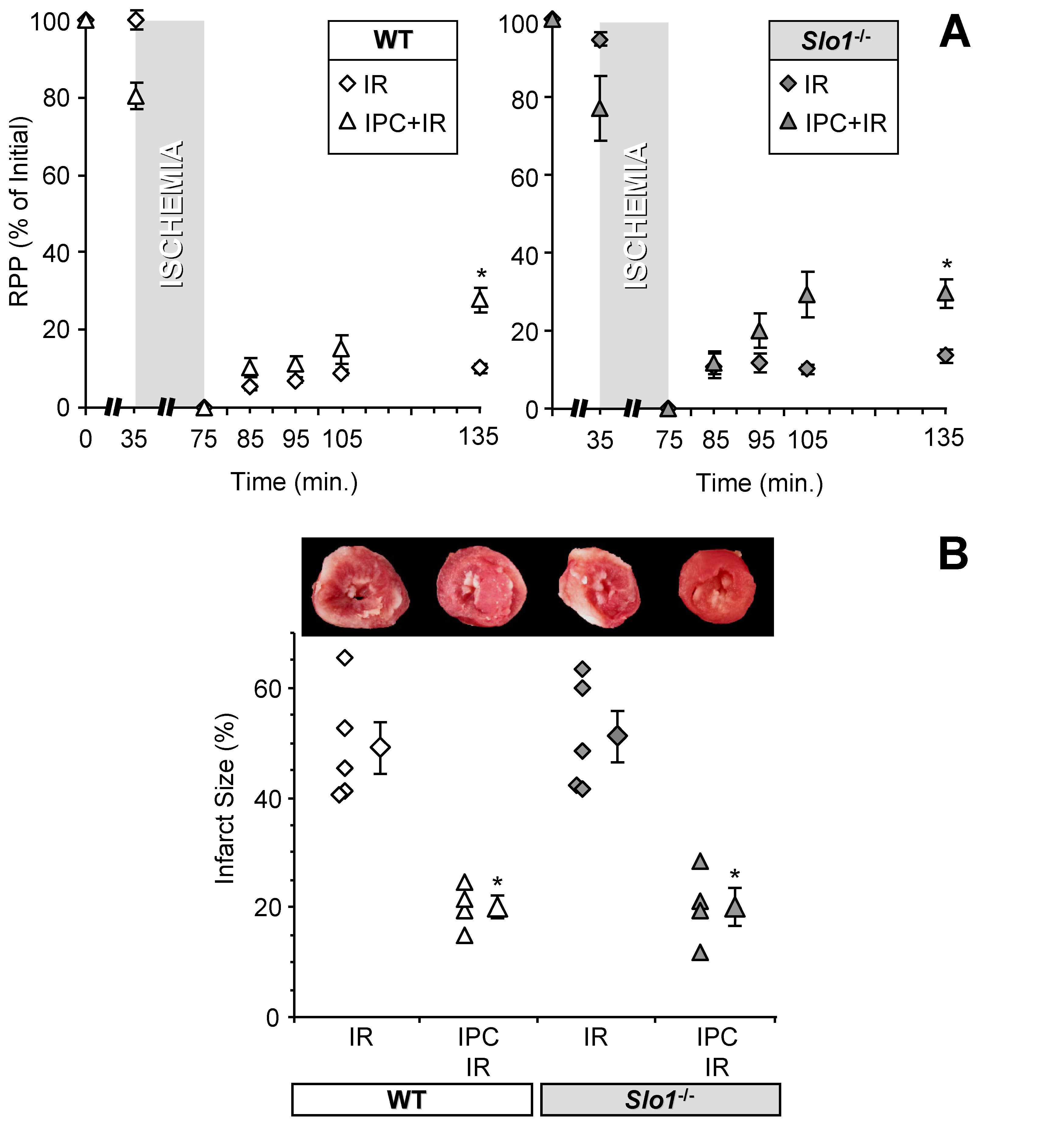

Supplement: Figure S1 — IPC in mouse hearts is independent of Slo1. Perfused hearts were subjected to IR injury (from Figure 2) or ischemic preconditioning (IPC)+IR, as outlined in Supporting Information S1 methods. (A) Left-ventricular function (heart rate x pressure product, RPP) was monitored throughout, and is expressed as % of initial value. Data for WT (white symbols) and Slo1-/- (gray symbols) FVB littermates are shown on separate axes for clarity. (B) Upon completion of IR protocols, hearts were sliced, fixed and stained with tetrazolium chloride, to delineate live (red) and infarcted (white) tissue. Upper panel shows typical slices used for quantitation of infarct area. Lower panel shows infarct expressed as a percent of the area at risk (100% in this global ischemia model). All data are means ± SEM, N≥4 (N = independent hearts).*p<0.05 vs. IR. (TIF) [file pone.0028287.s001.tif]

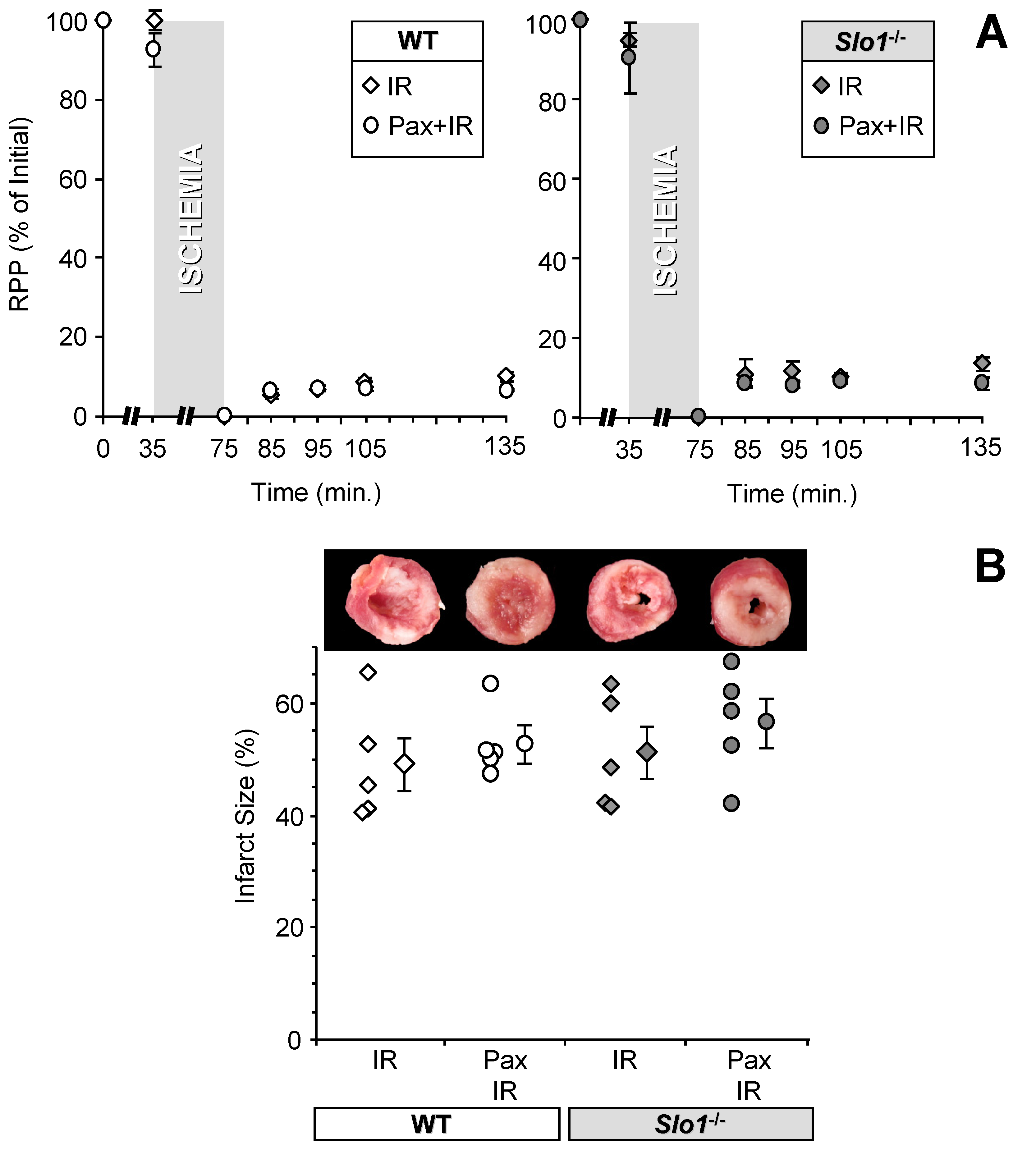

Supplement: Figure S2 — Paxilline in mouse hearts does not affect ischemic sensitivities. Perfused hearts were subjected to IR injury (from Figure 2) or paxilline (Pax)+IR, as outlined in Supporting Information S1 methods. (A) Left-ventricular function (heart rate x pressure product, RPP) was monitored throughout, and is expressed as % of initial value. Data for WT (white symbols) and Slo1-/- (gray symbols) FVB littermates are shown on separate axes for clarity. (B) Upon completion of IR protocols, hearts were sliced, fixed and stained with tetrazolium chloride, to delineate live (red) and infarcted (white) tissue. Upper panel shows typical slices used for quantitation of infarct area. Lower panel shows infarct expressed as a percent of the area at risk (100% in this global ischemia model). All data are means ± SEM, N = 5 (N = independent hearts). (TIF) [file pone.0028287.s002.tif]

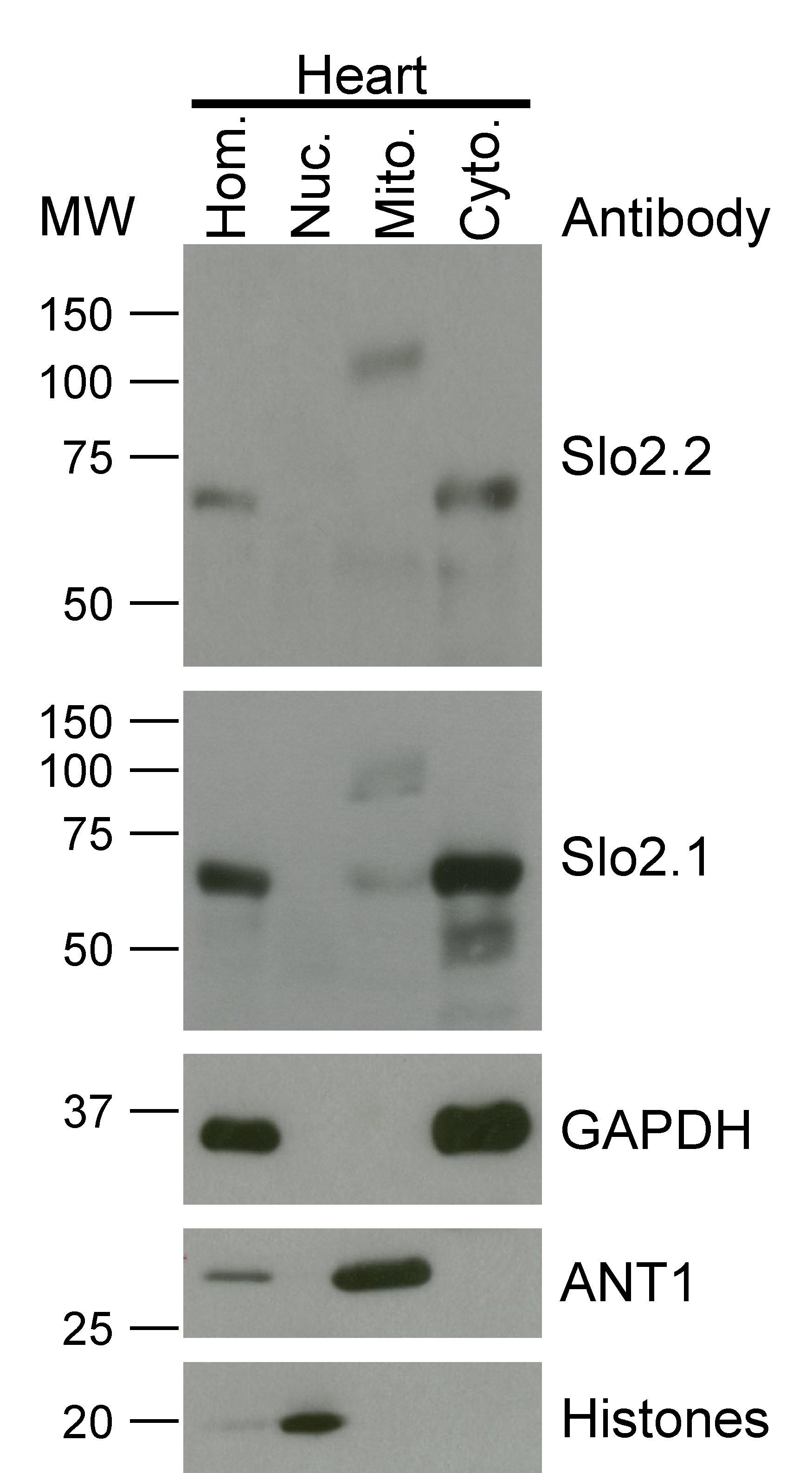

Supplement: Figure S3 — Immunoblot analysis of SLO2 in fractionated cardiac tissue. Homogenate from WT (C57BL/6) mouse hearts was fractionated and the proteins were separated by SDS-PAGE. Slo2.1 and Slo2.2 were detected by immunoblot analysis (NeuroMab antibodies), as detailed in Supporting Information S1 methods. Western blots for GAPDH, adenine nucleotide translocator 1 (ANT1) and histones validated separation of the homogenate into cytosolic, mitochondrial and nuclear fractions, respectively. (TIF) [file pone.0028287.s003.tif]

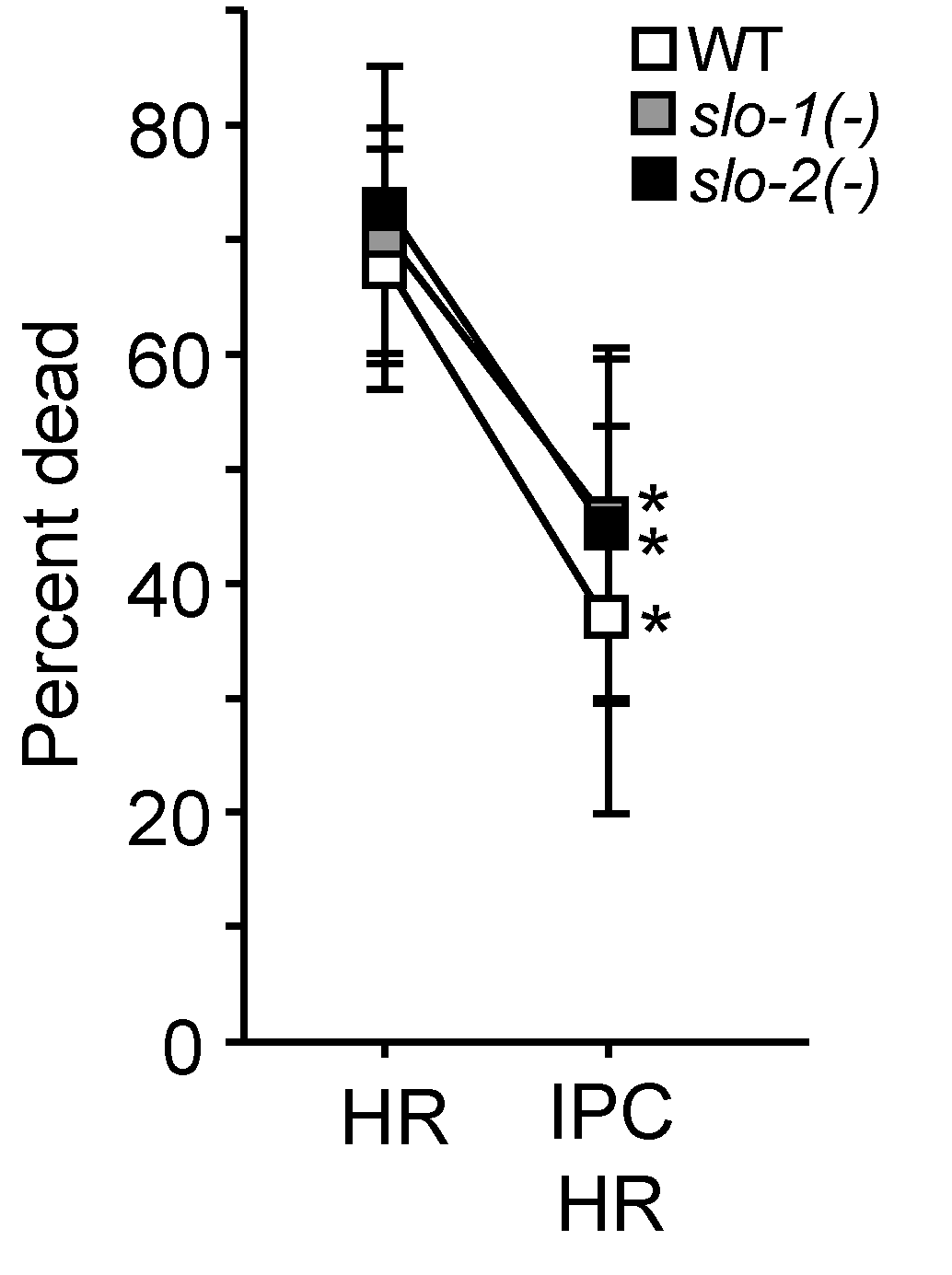

Supplement: Figure S4 — IPC in C. elegans is independent of slo-1 and slo-2. WT, slo-1(js379) and slo-2(nf100) mutants were subjected to hypoxia-reoxygenation (HR) and ischemic preconditioning IPC+HR, as detailed in the methods section of Supporting Information S1. Viability is expressed as percent of dead worms. Means ± SEM, N = 4 (N = independent trials of >100 worms per trial), *p<0.05 vs. HR. (TIF) [file pone.0028287.s004.tif]
