# Supplementary material for: SLO-2 Is Cytoprotective and Contributes to Mitochondrial Potassium Transport
Source: PLoS One. 2011 Dec 1;6(12):e28287. doi: 10.1371/journal.pone.0028287 (PMC3228735; doi:10.1371/journal.pone.0028287)
Supplement: Table S1 — Mitochondrial membrane potential is not affected by channel modulators. Mitochondria were isolated from WT (C57BL/6) mice and loaded with a fluorescent indicator (TMRE 20 nM or JC-1 0.2 µg/mL) in the presence of either Bithionol (2.5 µM), CaCl2 (25 µM), Paxilline (1 µM) or Bepridil (10 µM). Fluorescent indicators accumulate in mitochondria in relation to membrane potential (Δψm). Following stabilization, Δψm was collapsed via addition of Δψm FCCP (10 µM) resulting in a re-distribution of the fluorescent indicator, resulting in a decrease in fluorescence. All data are means ± SEM, N≥3 and are not significantly different (N = independent mitochondria isolation of ≥3 mouse hearts). (PDF) [file pone.0028287.s005.pdf]

Supplementary Tables:

Treatment	Δ Fluorescence (arbitrary units) TMRE	JC-1
Control	46.2 ± 4.8	2.63 ± 0.34
Bithionol (2.5 µM)	44.8 ± 4.8	3.01 ± 0.04
$CaCl_2$ (25 µM)	46.7 ± 4.9	2.59 ± 0.32
Paxilline (1 µM)	45.6 ± 4.4	2.88 ± 0.07
Bepridil (10 µM)	-	2.98 ± 0.12

Table S1. Mitochondrial membrane potential is not affected by channel modulators. Mitochondria were isolated from WT (C57BL/6) mice and loaded with a fluorescent indicator (TMRE 20 nM or JC-1 0.2 µg/mL) in the presence of either Bithionol (2.5 µM), $CaCl_2$ (25 µM), Paxilline (1 µM) or Bepridil (10 µM). Fluorescent indicators accumulate in mitochondria in relation to membrane potential ($\Delta\psi_m$). Following stabilization, $\Delta\psi_m$ was collapsed via addition of $\Delta\psi_m$ FCCP (10 µM) resulting in a re-distribution of the fluorescent indicator, resulting in a decrease in fluorescence. All data are means ± SEM, N≥3 and are not significantly different (N=independent mitochondria isolation of ≥3 mouse hearts).
