# Supplementary material for: SLO-2 Is Cytoprotective and Contributes to Mitochondrial Potassium Transport
Source: PLoS One. 2011 Dec 1;6(12):e28287. doi: 10.1371/journal.pone.0028287 (PMC3228735; doi:10.1371/journal.pone.0028287)
Supplement: Table S2 — EKG parameters of Avertin anesthetized wild-type (WT) and Slo1 -/- littermate FVB mice. EKG was collected as outlined in the methods. All data are means ± SEM, N≥13 *p<0.05 vs. WT. (PDF) [file pone.0028287.s006.pdf]

Supplementary Tables:

Parameter (units)	*FVB WT*	*FVB Slo1$^{-/-}$*
Heart Rate (BPM)	466 ± 7	429 ± 14*
PR interval (ms)	28 ± 0.5	28 ± 0.5
QRS diameter (ms)	17 ± 0.3	18 ± 0.5
Corrected QT interval (ms)	57 ± 0.8	56 ± 1.2

Table S2. EKG parameters of Avertin anesthetized wild-type (WT) and Slo1$^{-/-}$ littermate FVB mice. EKG was collected as outlined in the methods. All data are means ± SEM, N≥13 *p<0.05 vs. WT.
