# Supplementary material for: SLO-2 Is Cytoprotective and Contributes to Mitochondrial Potassium Transport
Source: PLoS One. 2011 Dec 1;6(12):e28287. doi: 10.1371/journal.pone.0028287 (PMC3228735; doi:10.1371/journal.pone.0028287)
Supplement: Table S3 — Mouse langendorff functional parameters. Groups are indicated in the left column in both wild-type (WT, top table) and Slo1 -/- (bottom table) FVB littermates. Parameters (indicated in the top row) were measured at the time points indicated. LVDP, left ventricular developed pressure (% of control). Data are means ± SEM, N≥4 *p<0.05 vs. IR (60 min Reperfusion). †p<0.05 vs. APC+IR (60 min Reperfusion). (PDF) [file pone.0028287.s007.pdf]

Supplementary Tables:

FVB WT

Group	Parameter	-dP/dT	+dP/dT	LVDP
IR	Baseline	-2549.3 ± 342.1	3337.6 ± 351.5	100
	Post-treatment	-2573.4 ± 349.5	3333.2 ± 377.9	102.8 ± 1.6
	30 min Reperfusion	-191.8 ± 37.1	160.0 ± 17.7	13.7 ± 0.9
	60 min Reperfusion	-170.0 ± 30.8	134.3 ± 27.1	14.2 ± 1.6
APC+IR	Baseline	-2517.5 ± 229.1	3821.2 ± 325.8	100
	Post-treatment	-2402.0 ± 125.8	3854.8 ± 193.3	112.0 ± 7.8
	30 min Reperfusion	-523.8 ± 124.9	496.7 ± 124.0	24.3 ± 4.7
	60 min Reperfusion	-582.2 ± 65.1*	645.5 ± 66.7*	31.5 ± 3.4*
Pax+APC+IR	Baseline	-2149.4 ± 201.5	3115.8 ± 308.9	100
	Post-treatment	-2109.4 ± 269.6	3198.4 ± 328.4	108.5 ± 5.6
	30 min Reperfusion	-174.0 ± 33.3	119.1 ± 21.9	10.8 ± 1.9
	60 min Reperfusion	-168.6 ± 44.2†	157.9 ± 36.0†	11.2 ± 1.7†
Pax+IR	Baseline	-2190.7 ± 153.5	3285.4 ± 200.4	100
	Post-treatment	-2235.9 ± 117.5	3491.2 ± 219.6	105.3 ± 3.9
	30 min Reperfusion	-157.8 ± 30.9	121.0 ± 11.5	11.5 ± 1.1
	60 min Reperfusion	-151.8 ± 29.3	122.3 ± 13.0	11.6 ± 0.9
Bt+IR	Baseline	-1780.3 ± 164.8	2657.3 ± 237.6	100
	Post-treatment	-2022.0 ± 337.4	2903.0 ± 306.5	105.1 ± 3.4
	30 min Reperfusion	-714.8 ± 158.0	689.0 ± 177.0	43.5 ± 10.3
	60 min Reperfusion	-842.0 ± 192.9*	985.8 ± 213.7*	53.9 ± 11.3*
IPC+IR	Baseline	-2211.1 ± 181.5	3107.7 ± 224.3	100
	Post-treatment	-1678.2 ± 204.4	2474.2 ± 249.0	89.2 ± 2.0
	30 min Reperfusion	-255.8 ± 67.1	357.7 ± 108.2	16.4 ± 3.3
	60 min Reperfusion	-534.3 ± 79.3*	728.9 ± 102.8*	29.4 ± 2.9*

Table S3. Mouse langendorff functional parameters. Groups are indicated in the left column in both wild-type (WT, top table) and *Slo1*$^{-/-}$ (bottom table) FVB littermates. Parameters (indicated in the top row) were measured at the time points indicated. LVDP, left ventricular developed pressure (% of control). Data are means ± SEM, N≥4 *p<0.05 vs. IR (60 min Reperfusion). †p<0.05 vs. APC+IR (60 min Reperfusion).

Supplementary Tables:

FVB Slo1$^{-/-}$

Group	Parameter	-dP/dT	+dP/dT	LVDP
**IR**	Baseline	-1982.8 ± 140.5	2903.6 ± 271.6	100
	Post-treatment	-2145.0 ± 267.9	3172.2 ± 373.9	100.9 ± 4.0
	30 min Reperfusion	-172.2 ± 40.0	117.4 ± 25.4	12.4 ± 2.6
	60 min Reperfusion	-170.3 ± 35.4	161.5 ± 30.6	15.6 ± 2.8
**APC+IR**	Baseline	-2438.9 ± 94.7	3682.8 ± 147.2	100
	Post-treatment	-2334.7 ± 147.8	3444.4 ± 215.9	98.8 ± 5.3
	30 min Reperfusion	-545.2 ± 75.9	634.8 ± 136.5	32.0 ± 5.1
	60 min Reperfusion	-664.5 ± 107.6*	785.9 ± 129.9*	37.0 ± 5.0*
**Pax+APC+IR**	Baseline	-2031.4 ± 90.4	3159.3 ± 169.8	100
	Post-treatment	-1855.8 ± 156.5	3037.2 ± 231.6	93.3 ± 4.3
	30 min Reperfusion	-162.3 ± 38.5	119.2 ± 12.7	12.1 ± 1.5
	60 min Reperfusion	-172.1 ± 24.7†	139.0 ± 24.1†	10.2 ± 0.9†
**Pax+IR**	Baseline	-1966.7 ± 76.7	2996.9 ± 119.7	100
	Post-treatment	-1976.6 ± 129.5	3064.3 ± 106.5	106.2 ± 1.9
	30 min Reperfusion	-200.1 ± 56.8	181.2 ± 56.2	14.0 ± 1.6
	60 min Reperfusion	-188.6 ± 43.3	196.4 ± 55.8	13.9 ± 2.1
**Bt+IR**	Baseline	-1675.6 ± 254.0	2868.8 ± 626.4	100
	Post-treatment	-1672.6 ± 110.3	2539.4 ± 194.0	107.2 ± 6.8
	30 min Reperfusion	-220.4 ± 36.1	302.5 ± 30.5	27.1 ± 2.7
	60 min Reperfusion	-540.6 ± 37.4*	749.8 ± 33.8*	35.7 ± 5.6*
**IPC+IR**	Baseline	-2003.5 ± 40.8	2762.1 ± 227.9	100
	Post-treatment	-1567.0 ± 131.9	2297.8 ± 265.5	88.6 ± 1.6
	30 min Reperfusion	-404.9 ± 130.1	649.1 ± 171.5	37.6 ± 9.7
	60 min Reperfusion	-730.1 ± 202.0*	1021.9 ± 287.2*	48.3 ± 11.3*

Table S3. Mouse langendorff functional parameters (continued).
