# Supplementary material for: SLO-2 Is Cytoprotective and Contributes to Mitochondrial Potassium Transport
Source: PLoS One. 2011 Dec 1;6(12):e28287. doi: 10.1371/journal.pone.0028287 (PMC3228735; doi:10.1371/journal.pone.0028287)
Supplement: Table S4 — APC-dependent protection in C. elegans requires slo-2. C. elegans WT control (N2-Bristol), slo-1(js379)V, slo-2(nf100)X, and slo-2(ok2214)X mutants were subjected to hypoxia-reoxygenation (HR) and isoflurane APC+HR, as detailed in the methods section of Supporting Information S1, and the average reduction in % death was determined. Means ± SEM. N2, N = 28; slo-1(js379), N = 12; slo-2(nf100), N = 27; slo-2(ok2214), N = 4. (N = independent trials of >100 worms per trial). (PDF) [file pone.0028287.s008.pdf]

Supplementary Tables:

C. elegans strain	*Percent Protected by APC*
N2	38 ± 0.5
slo-1(js379)	33 ± 1.6
slo-2(nf100)	19 ± 0.5
slo-2(ok2214)	8 ± 2.7

Table S4. APC-dependent protection in *C. elegans* requires *slo-2.* *C. elegans* WT control (N2-Bristol), *slo-1(js379)*V, *slo-2(nf100)*X, and *slo-2(ok2214)*X mutants were subjected to hypoxia-reoxygenation (HR) and isoflurane APC+HR, as detailed in the methods, and the average reduction in % death was determined. Means ± SEM. N2, N=28; *slo-1(js379)*, N=12; *slo-2(nf100)*, N=27; *slo-2(ok2214)*, N=4. (N= independent trials of >100 worms per trial).
