# Supplementary material for: SLO-2 Is Cytoprotective and Contributes to Mitochondrial Potassium Transport
Source: PLoS One. 2011 Dec 1;6(12):e28287. doi: 10.1371/journal.pone.0028287 (PMC3228735; doi:10.1371/journal.pone.0028287)
Supplement: Supporting Information S1 — Supplementary methods. (DOC) [file pone.0028287.s009.doc]

**Supporting Information for Wojtovich *et al.* “SLO-2 is Cytoprotective and Contributes to Mitochondrial Potassium Transport”**

**Supplementary Methods**

***C. elegans.***

Strains used in this study include Bristol-N2 (wild-type, WT); NM1968 *slo-1(js379)*V; LY100 *slo-2(nf100)*X; VC1819 *slo-2(ok2214)*X; KWN193 *pha-1(e2123)*III, *him-5(e1490)*V rnyEx112 [pKT60 (partial *Slo-2*::mCherry) recombined in vivo with linear WRM061CF07 fosmid, pCL1 (*pha-1*+)]; KWN 314 *slo-2(nf100)*X, *pha-1 (e2123ts)*III, rnyEx112; KWN 352 *pha-1(e2123)*III *him-5(e1490)*V, rnyEx216 [pKT111 (P*slo-2*::SLO-2::GFP), pCL1 (pha-1+)]. All mutant alleles were obtained from the *C. elegans* Genetic Center and were either obtained backcrossed or were backcrossed onto an N2-Bristol background. Single worm PCR genotyping was used to follow the mutant alleles.

***Mice.***

Characterization of the mBK channel was performed using male wild-type (WT) C57BL6 mice age 6-8 weeks purchased from The Jackson Laboratory (Bar Harbor, ME). Experiments involving *Kcnma1^tm1Rwa^* (*Slo1*^-/-^) knockout mice were on an FVB background [1]. Mice were genotyped by tail biopsy PCR, as previously described [1]. Male wild-type (WT) and *Slo1*^-/-^ littermates age 6-8 weeks were used in experiments. All mice were maintained in an AAALAC-accredited pathogen-free barrier facility with food and water available *ad libitum*. All procedures were in accordance with the NIH Guide for the Care and Use of Laboratory Animals and were approved by an Institutional Animal Care and Use Committee.

***Synchronized populations of worms for mitochondrial preparation.***

*C. elegans* were collected and precipitated from one 150 mm plate, and washed three time with M9 [2]. The *C. elegans* pellet was resuspended in 25 ml alkaline bleach solution (20% v/v bleach, 0.5 M NaOH) and centrifuged at 2000 x g for 1 min. The supernatant was removed and another 25 ml of alkaline bleach was added. The tube was inverted once every 15 s. until ~ 50% of *C. elegans* were lysed (total time < 5 min). The *C. elegans* were then centrifuged at 2000 x g for 1 min. The pellet was rinsed with 50 mL egg buffer (118 mM NaCl, 48 mM KCl, 2 mM CaCl_2_, 2 mM MgCl_2_, 25 mM HEPES, pH 7.3) and was centrifuged at 2000 x g for 1 min. This wash step was repeated 3 times. After counting, embryos were resuspended at 250,000/ml and plated at 0.8 ml/150 mm plate, each seeded with 6 ml of HB101 bacteria (~50% slurry), condensed from a 500 ml culture of Terrific Broth. Plates were incubated at 20°C for 3-4 days, resulting in staged adult *C. elegans* ready for mitochondrial isolation.

***Hypoxic killing and APC of C. elegans.***

Experiments were performed on chronologically-synchronized populations of young-adult animals. Briefly, adult hermaphrodites were allowed to lay embryos for a period of two hours, and their adult progeny were tested for the ability to be preconditioned essentially as described [3]. The following protocols were observed:

*Hypoxic killing.* *C. elegans* in M9 were incubated at 26ºC for 20 hrs. in an anoxic chamber (Coy Labs), containing 5% Hydrogen balanced with 95 % N_2_. After the hypoxic insult *C. elegans* were returned to normoxia at 20°C for 24 hrs. and scored. Animals without spontaneous or touch-evoked movement were scored as dead.

*APC.* *C. elegans* on uncovered NGM plates were placed in a small air tight chamber, as described [3]. A measured volume of liquid isoflurane was deposited into the chamber using a syringe, the chamber was quickly sealed, and the isoflurane was allowed to volatilize. The volume of isoflorane was calculated to produce a desired gas concentration of 2% final. Chambers containing anesthetic or air control were incubated for 4 hrs at 20°C then moved to atmospheric conditions at 20°C for one hour to allow for recovery. The preconditioned *C. elegans* were then subjected to hypoxic killing as above.

*IPC.* *C. elegans* were incubated for 4 hrs at 20°C in an anoxic chamber then moved to atmospheric conditions at 20°C for 20 hours to allow for recovery. The preconditioned *C. elegans* were then subjected to hypoxic killing as above. For all experiments, at least three replicates of fifty *C. elegans* were observed under paired normoxic/hypoxic conditions for each of the mutant strains.

***Fluorescent imaging.***

*C. elegans* expressing the fluorescent markers were placed onto 2% M9 agarose pads and examined using an Olympus FV1000 confocal microscope (available as part of the University of Rochester Confocal Core) under appropriate illumination.

***Isolation of mitochondria from C. elegans***

Mitochondria were isolated from *C. elegans* as previously described [2]. *C. elegans* from 4 plates of staged young adults (~250,000 *C. elegans* per 150 mm plate) were transferred at 25°C into 50 ml of M9 media (22 mM KH_2_PO_4_, 42 mM Na_2_HPO_4_, 86 mM NaCl, 1mM MgSO_4_, pH 7) in a conical tube. Worms were then precipitated by gravity on ice for 20 min. The supernatant was removed, and the pellet was rinsed twice with ice-cold M9 followed by a final rinse with 45 ml ice-cold mitochondrial isolation medium (IM: 220 mM mannitol, 70 mM sucrose, 5 mM MOPS, 2 mM EGTA, 0.4% BSA, pH 7.4, filtered). Worms were allowed to settle by gravity for 15 min. between rinses, and finally collected by centrifugation for 1 min at 1000 x g. The supernatant was removed leaving a dense pellet of worms. Worms were transferred 800 μl-1 ml at a time to a 4°C mortar (on ice) containing 2 g of washed sand, then ground with a pestle for 1 min and extracted from the sand by transferring to a 15 ml conical tube using three 3 ml aliquots of IM. To remove excess sand, worms were washed and allowed to settle three times using IM containing 0.4% BSA. The suspension was then homogenized with a glass Dounce homogenizer in IM. The homogenate was diluted and centrifuged at 600 x *g* for 5 min. The supernatant was then filtered through 300 µm mesh and centrifuged at 7000 x *g* for 10 min. The mitochondria enriched pellet was resuspended in 800 µL of IM and placed in a stirring cuvette for BTC-AM (a fluorescent Tl^+^ probe) loading. Four plates of *C. elegans* yielded ~12 mg mitochondrial protein.

***Isolation of mitochondria from mouse heart***

Mitochondria were isolated from mouse hearts as previously described [4]. Briefly, following anesthesia, 3 hearts were rapidly removed. and chopped into 2 mm cubes followed by homogenization (Polytron) in 3 ml HMIM (HMIM: 300 mM sucrose, 20 mM Tris, 2 mM EDTA, pH 7.35 at 4°C). The resulting homogenate was centrifuged at 2000 rpm for 5 min to pellet debris (Eppendorf desktop centrifuge 5417R). The supernatant was centrifuged at 10,000 rpm for 5 min resulting in a mitochondrial fraction. The mitochondria enriched pellet was resuspended in 800 µL of HMIM and placed in a stirring cuvette for BTC-AM loading. As previously reported for the K_ATP_ channel [5,6], rapid and somewhat crude mitochondrial preparation was necessary to preserve mitochondrial K^+^ channel function.

***BTC-AM loading.***

The mitochondria were incubated with 20 µM BTC-AM (Benzothiazole coumarin acetyoxymethyl ester; Invitrogen, B6791). and 0.05% Pluronic F-127 for 10 min at room temperature. Following incubation the mitochondria were washed twice with isolation medium (HMIM for mouse mitochondria; IM for *C. elegans* mitochondria) by centrifugation at 10,000 rpm for 5 min. The final mitochondrial pellet was suspended in 225 µl of the respective isolation medium and stored on ice until use, within 1.5 hrs. Protein was determined by the Folin-phenol method [7].

***mBK thallium flux assay (Tl^+^-flux).***

Tl^+^ is widely used as a surrogate for K^+^ and the Tl^+^ flux assay as a measue of K^+^ channel activity is widely validated in the cellular K^+^ channel field [8,9,10,11,12]. The application to mitochondrial K^+^ channels was recently described [13] and relies on the same principles and reagents. Changes in fluorescence due to Tl^+^ uptake in mitochondria were monitored at 525 nm using a Varian Cary Eclipse spectrofluorometer as previously described [13]. In the assay, ~0.8 mg/ml BTC-AM-loaded worm or ~0.2mg mouse mitochondria were added to a rapidly stirred cuvet containing 2 ml of Tl^+^ assay buffer (195 mM mannitol, 10 mM HEPES, 2 mM MgCl_2_, 2 mM Na_2_HPO_4_, and 1 µg/ml oligomycin, pH 7.2 at 25°C (*C. elegans*) or 37°C (mouse)) and mitochondrial substrate (2 mM pyruvate + 2 mM malate for *C. elegans*, or 2 mM succinate for mouse mitochondria). Compounds were incubated with mitochondria for 30 s., followed by recording 10 s. of baseline fluorescence. Tl_2_SO_4_ (2 mM) was added via a syringe port and the fluorescence was monitored (λ_ex_ 488 nm, λ_em_ 525 nm) and normalized to baseline.

***Mitochondrial membrane potential measurements.***

Mitochondria were isolated from 3 mouse hearts as described for BTC-AM loading omitting BTC-AM specific steps. Mitochondrial membrane potential (Δψ_m_) was monitored using TMRE (Ex 555 nm, Em 577 nm) or JC-1 (Ex 485, Em 530 & 590 nm) and a Varian Cary Eclipse spectrofluorometer. In the assay, ~0.2mg mouse mitochondria were added to a rapidly stirred cuvet containing 2 ml of Tl^+^ assay buffer (195 mM mannitol, 10 mM HEPES, 2 mM MgCl_2_, 2 mM Na_2_HPO_4_, 2 mM succinate, and 1 µg/ml oligomycin, pH 7.2 at 37°C). Compounds were incubated with mitochondria for 30 s., followed by recording 10 s. of baseline fluorescence. TMRE (20 nM) or JC-1 (0.2 μg/mL) was then added. Following stabilization, FCCP (10 μM) was added, resulting in the re-distribution of the fluorescent indicator and a decrease in fluorescence. The change in fluorescence upon FCCP addition is expressed in arbitrary fluorescence units for TMRE, and as a change in the ratio of J-aggregate (fluorescence at 590 nm) to monomer (fluorescence at 530 nm) for JC-1. Due to the lack of K^+^ in the Tl^+^ assay buffer, the channel activator bithionol did not result in a change in membrane potential associated with K^+^ influx.

***Immunoblotting of cell fractions.***

Cardiac tissue from WT (C57BL/6) mice was fractionated [14] and 30 μg of protein was separated by SDS-PAGE and transferred to nitrocellulose as previously described [15]. The primary antibodies were anti-Slo2.1 and anti-Slo2.2 (UC Davis/NIH NeuroMab Facility; 1:1000), anti-GAPDH (Chemicon, Billerica, MA; 1:20,000), anti-ANT1 (MitoScience-Abcam, Cambridge, MA; 1:25,000), anti-histone (Chemicon, Billerica, MA; 1:1000). Blots were developed with horseradish peroxidase-linked secondary antibodies and enhanced chemiluminescence [15].

***Mouse Langendorff ex-vivo perfused heart.***

Following anesthesia with avertin (100mg/kg IP), a rodent 3-lead EKG was briefly obtained on WT and *Slo1^-/-^* FVB littermates. QT_corr_ was calculated as [QT / (RR interval / 100)1/2] [16]. A thoracotomy was then performed, and the aorta cannulated *in-situ* with a 22½G needle filled with 37°C Krebs-Henseleit buffer (KH: 118 mM NaCl, 4.7 mM KCl, 25 mM NaHCO_3_, 10 mM glucose, 1.2 mM MgSO_4_, 1.2 mM KH_2_PO_4_, 2.5 mM CaCl_2_, bubbled with 95% O_2_, 5% CO_2_, 37°C) and rapidly transferred to a perfusion apparatus, as previously described [13]. The heart was perfused with KH buffer using constant flow (4 ml/min/100mg). Coronary root pressure was measured by a transducer in the cannula connected to a digital recording system (DATAQ). Left ventricular pressure was monitored using a transducer/DATAQ system connected to a water-filled balloon inserted into the left ventricle. The initial diastolic pressure was set to 5-10 mmHg by inflating the balloon. The following protocols were observed: 1) IR Injury: 35 min. normoxic perfusion, 40 min. of global ischemia, 60 min. reperfusion. 2) APC: 3 x 5 min. 300 µM isoflurane plus 5 min. washout, then IR as above. 3) APC + Paxilline: 35 min. of constant 1 µM paxilline during the APC protocol, 30 sec. washout then IR as above. 4) Paxilline: 35 min. of 1 µM paxilline, 30 sec. washout then IR as above. 5) IPC: 3 x 5 min. ischemia plus 5 min. perfusion, then IR as above. 6) Bithionol: 3 x 5 min. 10 nM Bithionol plus 5 min. washout, then IR as above. Paxilline stock solutions were prepared fresh in DMSO at 50 mM every week. When stored in solution for over a week, paxilline broke down into a cardiotoxic compound, as evidenced by a decrease in left ventricular developed pressure. Working stocks of paxilline were made up fresh in 1:1 water/ethanol. Paxilline and isoflurane were delivered via a syringe pump linked to an injection port immediately above the perfusion cannula. The volume of added solutions was ≤0.075% of the total perfusate volume.

***Quantitation of myocardial infarct size.***

Hearts were stained and imaged as previously described [15]. Briefly, following perfusion the heart was sliced transversely into 2 mm slices and stained in 1 % (w/v) 2,3,5-triphenyltetrazolium chloride (TTC) in 100 mM phosphate buffer (pH 7.4, 37°C) for 20 min. Slices were fixed in 10% neutral buffered formalin for 24 hr. to increase contrast. Images were collected by digital photography using a Canon Powershot SD990-IS (14.7 mega-pixels) under diffused lighting. Images were analyzed using Adobe Photoshop. The background color was set to black (R:0, G:0, B:0, tolerance 100), the infarcted tissue green (R:0, G:255, B:0, tolerance 140), and the live tissue red (R:255, G:0, B:0, tolerance 100) using a reference file of pure white (R:255, G:255, B:255) as the standard for infarcted tissue. The histogram tool was then used to quantify the number of pixels of each color. Infarct (green) vs. live (red) tissue was expressed as a percent of area at risk (100% in this global ischemia model).

***Reagents.***

All chemicals were of the highest grade available from Sigma (St. Louis, MO) unless otherwise specified. Iberiotoxin, Charybdotoxin, and Apamin were from EMD Chemicals Group (Darmstadt, Germany); Bepridil and Paxilline were from Enzo Life Sciences International, Inc. (Plymouth Meeting, PA); Bithionol was from TCI America (Portland, OR).

***Statistics.***

Data presented are mean ± SEM. Statistical significance (P<0.05) between multiple groups was determined using analysis of variance (ANOVA). In whole worm studies, significance (P<0.05) was determined using a paired Student’s *t*-test.

**REFERENCES**

1. Meredith AL, Thorneloe KS, Werner ME, Nelson MT, Aldrich RW (2004) Overactive bladder and incontinence in the absence of the BK large conductance Ca2+-activated K+ channel. J Biol Chem 279: 36746-36752.

2. Wojtovich AP, Burwell LS, Sherman TA, Nehrke KW, Brookes PS (2008) The C. elegans mitochondrial K+(ATP) channel: a potential target for preconditioning. Biochem Biophys Res Commun 376: 625-628.

3. Jia B, Crowder CM (2008) Volatile anesthetic preconditioning present in the invertebrate Caenorhabditis elegans. Anesthesiology 108: 426-433.

4. Nadtochiy SM, Burwell LS, Ingraham CA, Spencer CM, Friedman AE et al. (2009) In vivo cardioprotection by S-nitroso-2-mercaptopropionyl glycine. J Mol Cell Cardiol 46: 960-968.

5. Wojtovich AP, Brookes PS (2008) The endogenous mitochondrial complex II inhibitor malonate regulates mitochondrial ATP-sensitive potassium channels: implications for ischemic preconditioning. Biochim Biophys Acta 1777: 882-889.

6. Wojtovich AP, Brookes PS (2009) The complex II inhibitor atpenin A5 protects against cardiac ischemia-reperfusion injury via activation of mitochondrial KATP channels. Basic Res Cardiol 104: 121-129.

7. Lowry OH, Rosenbrough NJ, Farr AL, Randall RJ (1951) Protein measurement with the Folin phenol reagent. J Biol Chem 193: 265-275.

8. Weaver CD, Harden D, Dworetzky SI, Robertson B, Knox RJ (2004) A thallium-sensitive, fluorescence-based assay for detecting and characterizing potassium channel modulators in mammalian cells. J Biomol Screen 9: 671-677.

9. Beacham DW, Blackmer T, O' GM, Hanson GT (2010) Cell-based potassium ion channel screening using the FluxOR assay. J Biomol Screen 15: 441-446.

10. Bhave G, Chauder BA, Liu W, Dawson ES, Kadakia R et al. (2011) Development of a selective small-molecule inhibitor of Kir1.1, the renal outer medullary potassium channel. Mol Pharmacol 79: 42-50.

11. Niswender CM, Johnson KA, Luo Q, Ayala JE, Kim C et al. (2008) A novel assay of Gi/o-linked G protein-coupled receptor coupling to potassium channels provides new insights into the pharmacology of the group III metabotropic glutamate receptors. Mol Pharmacol 73: 1213-1224.

12. Hougaard C, Eriksen BL, Jorgensen S, Johansen TH, Dyhring T et al. (2007) Selective positive modulation of the SK3 and SK2 subtypes of small conductance Ca2+-activated K+ channels. Br J Pharmacol 151: 655-665.

13. Wojtovich AP, Williams DM, Karcz MK, Lopes CM, Gray DA et al. (2010) A Novel Mitochondrial KATP Channel Assay. Circ Res 106: 1190-1196.

14. Nadtochiy SM, Yao H, McBurney MW, Gu W, Guarente LP et al. (2011) SIRT1 mediated acute cardioprotection. Am J Physiol Heart Circ Physiol

15. Nadtochiy SM, Redman E, Rahman I, Brookes PS (2011) Lysine deacetylation in ischaemic preconditioning: the role of SIRT1. Cardiovasc Res 89: 643-649.

16. Korte T, Fuchs M, Guener Z, Bonin J, de SM et al. (2002) In-vivo electrophysiological study in mice with chronic anterior myocardial infarction. J Interv Card Electrophysiol 6: 121-132.

**Supplementary Figures:**

**Figure S1. IPC in mouse hearts is independent of *Slo1.*** Perfused hearts were subjected to IR injury (from Figure 2) or ischemic preconditioning (IPC)+IR, as outlined in the methods section of the Supporting Information. (A) Left-ventricular function (heart rate x pressure product, RPP) was monitored throughout, and is expressed as % of initial value. Data for WT (white symbols) and *Slo1^-/-^* (gray symbols) FVB littermates are shown on separate axes for clarity. (B) Upon completion of IR protocols, hearts were sliced, fixed and stained with tetrazolium chloride, to delineate live (red) and infarcted (white) tissue. Upper panel shows typical slices used for quantitation of infarct area. Lower panel shows infarct expressed as a percent of the area at risk (100 % in this global ischemia model). All data are means ± SEM, N≥4 (N=independent hearts).*p<0.05 vs. IR.

**Figure S2. Paxilline in mouse hearts does not affect ischemic sensitivities*.*** Perfused hearts were subjected to IR injury (from Figure 2) or paxilline (Pax)+IR, as outlined in the methods section of the Supporting Information. (A) Left-ventricular function (heart rate x pressure product, RPP) was monitored throughout, and is expressed as % of initial value. Data for WT (white symbols) and *Slo1^-/-^* (gray symbols) FVB littermates are shown on separate axes for clarity. (B) Upon completion of IR protocols, hearts were sliced, fixed and stained with tetrazolium chloride, to delineate live (red) and infarcted (white) tissue. Upper panel shows typical slices used for quantitation of infarct area. Lower panel shows infarct expressed as a percent of the area at risk (100 % in this global ischemia model). All data are means ± SEM, N=5 (N= independent hearts).

**Figure S3.** **Immunoblot analysis of SLO2 in fractionated cardiac tissue.** Homogenate from WT (C57BL/6) mouse hearts was fractionated and the proteins were separated by SDS-PAGE. Slo2.1 and Slo2.2 were detected by immunoblot analysis (NeuroMab antibodies), as detailed in the Supporting Information methods. Western blots for GAPDH, adenine nucleotide translocator 1 (ANT1) and histones validated separation of the homogenate into cytosolic, mitochondrial and nuclear fractions, respectively.

**Figure S4. IPC in *C. elegans* is independent of *slo-1* and *slo-2.*** WT, *slo-1(js379)* and *slo-2(nf100)* mutants were subjected to hypoxia-reoxygenation (HR) and ischemic preconditioning IPC+HR, as detailed in the methods section of the Supporting Information. Viability is expressed as percent of dead worms. Means ± SEM, N=4 (N=independent trials of >100 worms per trial), *p<0.05 vs. HR.

**Supplementary Tables:**

**Table S1. Mitochondrial membrane potential is not affected by channel modulators.** Mitochondria were isolated from WT (C57BL/6) mice and loaded with a fluorescent indicator (TMRE 20 nM or JC-1 0.2 μg/mL) in the presence of either Bithionol (2.5 μM), CaCl_2_ (25 μM), Paxilline (1 μM) or Bepridil (10 μM). Fluorescent indicators accumulate in mitochondria in relation to membrane potential (Δψ_m_). Following stabilization, Δψ_m_ was collapsed via addition of Δψ_m_ FCCP (10 μM) resulting in a re-distribution of the fluorescent indicator, resulting in a decrease in fluorescence. All data are means ± SEM, N≥3 and are not significantly different (N=independent mitochondria isolation of ≥3 mouse hearts).

**Table S2. EKG parameters of Avertin anesthetized wild-type (WT) and *Slo1*^-/-^ littermate FVB mice**. EKG was collected as outlined in the methods. All data are means ± SEM, N≥13 *p<0.05 vs. WT.

**Table S3. Mouse langendorff functional parameters.** Groups are indicated in the left column in both wild-type (WT, top table) and *Slo1*^-/-^ (bottom table) FVB littermates. Parameters (indicated in the top row) were measured at the time points indicated. LVDP, left ventricular developed pressure (% of control). Data are means ± SEM, N≥4 *p<0.05 vs. IR (60 min Reperfusion). †p<0.05 vs. APC+IR (60 min Reperfusion).

**Table S4. APC-dependent protection in *C. elegans* requires *slo-2.*** *C. elegans* WT control (N2-Bristol), *slo-1(js379)*V, *slo-2(nf100)*X, and *slo-2(ok2214)*X mutants were subjected to hypoxia-reoxygenation (HR) and isoflurane APC+HR, as detailed in the methods section of the Supporting Information, and the average reduction in % death was determined. Means ± SEM. N2, N=28; *slo-1(js379)*, N=12; *slo-2(nf100)*, N=27; *slo-2(ok2214)*, N=4. (N= independent trials of >100 worms per trial).
